# A novel rapid detection approach based on CRISPR-Cas13a for *Dermatophagoides pteronyssinus* and *Dermatophagoides farinae* (Acariformes: Pyroglyphidae)

**DOI:** 10.1093/jisesa/ieag029

**Published:** 2026-04-18

**Authors:** Siwen Liu, Lan Wang, Hong Zhang, Feng Jiang, Feifan Tang, Renren Han, Wei Guo, Shengli Gu, Guoqing Chen, Dacun Zhang, Xiaodong Zhan

**Affiliations:** Department of Medical Parasitology, Wannan Medical University, Wuhu, Anhui, China; Department of Medical Parasitology, Wannan Medical University, Wuhu, Anhui, China; Auhui Provincial Engineering Research Centre for Molecular Detection and Diagnostics, Anhui Normal University, Wuhu, China; Wuhu 3h Biotechnology Co., Ltd, Wuhu, China; Department of Medical Parasitology, Wannan Medical University, Wuhu, Anhui, China; Wuhu Mites Inspection and Control Engineering Technology Research Center, Wuhu, China; Anhui Province Key Laboratory of Basic Research and Transformation of Age-related Diseases, Wuhu, China; Department of Medical Parasitology, Wannan Medical University, Wuhu, Anhui, China; Department of Medical Parasitology, Wannan Medical University, Wuhu, Anhui, China; Department of Medical Parasitology, Wannan Medical University, Wuhu, Anhui, China; Wuhu Mites Inspection and Control Engineering Technology Research Center, Wuhu, China; Anhui Province Key Laboratory of Basic Research and Transformation of Age-related Diseases, Wuhu, China; Department of Medical Parasitology, Wannan Medical University, Wuhu, Anhui, China; Wuhu Mites Inspection and Control Engineering Technology Research Center, Wuhu, China; Anhui Province Key Laboratory of Basic Research and Transformation of Age-related Diseases, Wuhu, China; Shandong Jiuxin Bioengineering Co., Ltd, Shandong, China; Shandong Jiuxin Bioengineering Co., Ltd, Shandong, China; Department of Medical Parasitology, Wannan Medical University, Wuhu, Anhui, China; Wuhu Mites Inspection and Control Engineering Technology Research Center, Wuhu, China; Anhui Province Key Laboratory of Basic Research and Transformation of Age-related Diseases, Wuhu, China

**Keywords:** Molecular diagnostics, Allergies, Environmental allergen testing

## Abstract

*Dermatophagoides pteronyssinus* (Trouessart) and *Dermatophagoides farinae* (Hughes) (Acariformes: Pyroglyphidae) are the prevalent kinds of house dust mites (HDMs). HDM is a common indoor pest, which mainly breeds in indoor dust and is an important allergen source causing a variety of allergic diseases. Effective detection of these HDMs is crucial in preventing the allergic diseases they cause. The objective of this study was to develop an innovative method for the rapid visualization of HDMs (*D. pteronyssinus* and *D. farinae*) using recombinase polymerase amplification (RPA) and lateral flow dipstick (LFD) in combination with CRISPR-Cas13a (RPA–Cas13a–LFD). To achieve heightened sensitivity in the detection of HDMs, Cas13a was incorporated into the RPA process and coupled with T7 transcripts. Based on this approach, a total of 2.23–10^2^ copies/μl of HDM were detected within 35 min (detection limit of 2.23 copies/μl for *D. farinae* and 39.7 copies/μl for *D. pteronyssinus*). No cross-reactivity occurred with *Aleuroglyphus ovatus* (Troupeau) (Acariformes: Acaridae), *Tyrophagus putrescentiae* (Schrank) (Acariformes: Acaridae), *Blomia tropicalis* (van Bronswijk, de Cock & Oshima) (Acariformes: Acaridae), *Suidasia nesbitti* (Hughes) (Acariformes: Acaridae), and *Carpoglyphus lactis*(Linnaeus) (Acariformes: Acaridae). The RPA–Cas13a–LFD methodology demonstrated high specificity and sensitivity in detecting HDM. Given its advantages, such as ease of operation, rapid detection, and time efficiency, it is well-suited for rapid field-based detection of HDMs, providing a new technical tool for detecting *D. farinae* and *D. pteronyssinus*.

## Introduction

House dust mite (HDM) is a mite that mainly breeds in indoor dust, and is common in carpets, beds, textiles, and furniture. The most common are *Dermatophagoides farinae* (Hughes) and *Dermatophagoides pteronyssinus* (Trouessart) (Acariformes: Pyroglyphidae) (Gómez et al. 1990). In 1964, Voorhorst and Spieksma (Voorhorst et al. 1967) first confirmed that HDM was the main source of allergens in household dust. HDM allergens (especially the allergens of house dust mite *D. pteronyssinus*) can cause allergic reactions and asthma complications; When its concentration increases, it poses a significant risk of acute asthma for susceptible individuals (Gómez et al. 1990). In addition, HDM is also associated with the pathogenesis of allergic rhinitis (Demoly et al. 2021) and contact dermatitis (Sunderkotter et al. 2021). According to statistics, HDM allergens account for about 1% to 2% of the total amount of environmental allergens, and are estimated to affect about 650 to 130 million people worldwide (Miller 2019). Allergic diseases usually involve multiple organ systems and have specific genetic susceptibility (Gans and Gavrilova 2020).

The main prevention and treatment measures include patient education (avoiding contact with allergens, rational drug use) and cleaning the home environment to reduce the concentration of allergens. Therefore, it is very important to establish a rapid and accurate detection method of HDM for the effective prevention and treatment of allergic diseases caused by HDM.

Current methods for detecting HDM are categorized into 2 principal types based on their detection mode: (i) molecular detection: which relies on RNA nucleic acid detection, primarily through methods such as reverse transcription polymerase chain reaction (RT-PCR). Although this method is practical, it takes a long time, requires professional knowledge and expensive instruments and equipment, and its application is limited in areas with limited resources; (ii) immunoassay: it is mainly based on antigen antibody reaction. Common methods include enzyme-linked immunosorbent assay (ELISA) and colloidal gold immunochromatography. Therefore, this method relies on antibodies produced by the human immune system, and is mainly used for screening, treatment effect, and rehabilitation status evaluation of sensitized people.

RNA is inherently more susceptible to degradation than DNA due to its single-stranded structure, but it also has clinically solid relevance for efficient assessment and prompt diagnosis. Different genes can be used to design qPCR assays to quantify gene copy number, such as mitochondrial large subunit ribosomal RNA (mtLSU rRNA) (Sun et al. 2025), mitochondrial Small Subunit Ribosomal RNA (mtSSU rRNA) (Rahman et al. 2013), nicotinamide adenine dinucleotide (NAD1) (Forero-Baena et al. 2015), and cytochrome b (CYTB) (Hadi et al. 2026). Previous research has shown that quantitative reverse transcriptase detection of RNA is more sensitive than other techniques and targets (Valero et al. 2016, Delliere et al. 2021). While recombinase polymerase amplification (RPA) and loop-mediated isothermal amplification (LAMP) are limited to DNA amplification, transcription-mediated amplification technology has been reported to amplify RNA effectively (Ma et al. 2020). However, this RNA-specific amplification approach has not been employed for dust mite detection.

In contrast to Cas12a, the usage of Cas13a incorporates an additional, yet manageable, transcription step. This transcription step entails a secondary amplification of the PCR amplicon, resulting in heightened sensitivity and a lower limit of detection (LOD). Moreover, Cas13a displays a more comprehensive linear detection range and a more favorable signal-to-background ratio compared to Cas12a, rendering it more effective in trans-cleavage and collateral cleavage processes (Zhou et al. 2020). Furthermore, previous studies have shown that at low activator concentrations, LbuCas13a achieves a markedly faster detection rate than LbCas12a (Chandrasekaran et al. 2022). Thus far, most CRISPR-based diagnostic methods have predominantly relied in the pre-amplification of the target, with RPA being the most frequently utilized isothermal amplification technique. The SHERLOCK platform, which combines RPA and CRISPR-Cas13a, has been extensively used to detect viruses, bacteria, and parasites. For instance, Wang et al. (2023) utilized RPA–Cas13a–LFD technology to rapidly detect porcine circovirus (PCV4) within 1.5 h, achieving a detection threshold of 1 × 10^0^ ng/μl and demonstrating 100% agreement with RT-PCR methodology in diagnostic performance. Within the field of parasitology, timely and accurate diagnosis is essential for efficacious healthcare interventions. A significant portion of the global population—nearly 47%—has limited or no access to medical diagnosis (Fleming et al. 2021). Therefore, rapid, accurate, field-operable, and cost-effective diagnostic tools are urgently needed to manage, control, and ultimately eliminate parasitic infections, thereby improving patient treatment outcomes.

The Clustered Regularly Interspaced Short Palindromic Repeat (CRISPR) system is a natural adaptive immune mechanism in most bacterial (about 40%) and archaeal species (about 90%) (Hille et al. 2018, Chen et al. 2019). Cas13a belongs to the vi-a protein of CRISPR/CAS family, and is a CRISPR effector targeting single-stranded RNA (ssRNA). Under the guidance of CRISPR RNA (crRNA), cas13a can not only cut target ssRNA but also nonspecifically cut other ssRNA molecules (Abudayyeh et al. 2016, Shmakov et al. 2017). This nonspecific cleavage ability of cas13a is due to its pararnase activity (collateral RNAse activity). The detection of this activity is usually carried out by using RNA substrates labeled with quenching fluorescent groups (called report RNA), which is realized by the change of fluorescent signal (East-Seletsky et al. 2016).

Recent years have seen isothermal amplification techniques, including cross primer amplification (Gans et al. 2020), LAMP (Park 2022), and recombinase-aided amplification (RPA) emerge as valuable methods for viral nucleic acid detection. RPA is also widely used due to its selectivity and rapid amplification of target DNA to detectable concentrations (Dong et al. 2020, Li et al. 2020).

The RPA–lateral flow dipstick (RPA–LFD) combination has proven effective for detecting diverse pathogens, including: *Salmonella typhimurium* (Salmonella enterica subsp. enterica serovar Typhimurium) (Enterobacterales: Enterobacteriaceae) (Onchan et al. 2022), *Mycoplasma pneumoniae* (Mycoplasma pneumoniae Somerson et al.,) (*Mycoplasmatales: Mycoplas­mataceae*) (Ma et al. 2023) and *Phytophthora sojae* (Kaufmann & Gerdemann) (Peronosporales: Pythiaceae) (Liu et al. 2023), Foot-and-mouth disease virus serotypes A, O, and Asia1 (Wang et al. 2018). Integrating recombinase-based isothermal amplification with Cas13a collateral cleavage enabled development of Specific High-sensitivity Enzymatic Reporter unLOCKing (SHERLOCK). This isothermal platform detects DNA and RNA targets with amore sensitivity and single-base mismatch specificity (Gootenberg et al. 2017). SHERLOCK has demonstrated efficacy in field-based pathogen detection, such as *Flavivirus* (Barnes et al. 2020) and *Ebolavirus* (Wang et al. 2022). When coupled with lateral flow test strips, it provides a practical visual readout for field environments.

This study aims to develop a novel detection method that integrates CRISPR-Cas13a with RPA–LFD technology. By targeting the major allergen genes (*Der f 1* and *Der p 1*) of dust mites, this method can quickly identify the presence of house dust mites (*D. farinae* and *D. pteronysinus*) and infer the main allergens that may trigger allergic reactions. Its goal is to improve the sensitivity and specificity of detection, while reducing the need for complex equipment and professional skills, thereby supporting early prevention and management of allergic diseases. The feasibility and LOD this method will be validated through experiments. Its performance will be compared with traditional qPCR and ELISA methods to demonstrate its superiority and potential for application.

## Materials and Methods

### Samples

Test participants can directly collect about 100 mg of dust samples from indoor environments (such as bed sheets, carpets, pillows, etc.). The collected dust samples do not need further processing and can be sent directly to the laboratory. *D. farinae* and *D. pteronyssinus* were randomly sampled from floor dust and cultured in an incubator at 25 ± 1 °C and relative humidity of 75% ± 5%. The culture medium comprised specific nutrient compositions: 33.3% dry yeast, 33.3% whole wheat flour, 16.7% wheat germ, and 16.7% dry fish meal for *D. farinae*; and 5% dried daphnia, 15% commercial goldfish food, 40% granulated yeast, and 40% wheat germ for *D. pteronyssinus* (Nam et al. 2007). *Aleuroglyphus ovatus* (Acariformes: Acaridae), *Tyrophagus putrescentiae* (Acariformes: Acaridae), *Blomia tropicalis* (Acariformes: Acaridae), *Suidasia nesbitti* (Acariformes: Acaridae), and *Carpoglyphus lactis* (Acariformes: Acaridae) were obtained from our laboratory and used to detect specificity.

### DNA Extraction

Firstly, the mites from the incubator were selected out from *D. farinae* and *D. pteronyssinus* using a microscope. Under the microscope, the body of the *D. pteronyssinus* is elongated oval. The female has a longitudinal striation in the center of the back. Leg III is relatively thick and long, while leg IV is short and small. The male’s posterior shield is longer than it is wide, legs I and II are of similar thickness, and the inner projections of segment I do not meet. The body of the *D. farinae* is elliptical, with the female showing transverse striations in the center of the back, and the end is arched. Legs III and IV are of similar thickness. The male’s posterior shield is short and wide, leg I is robust, and the inner projections of segment I meet, cleaned and put into 0.2-ml Eppendorf tube, respectively, and the DNA of the samples were extracted according to the A MolPure Cell/Tissue DNA Kit (Yeasen Biotechnology Co., Ltd., Shanghai, China): add 30 μl of buffer ATL (Yeasen Biotechnology Co., Ltd.), pipette to disperse evenly, wash the pipette tip with 30 μl of grinding buffer, add 20 μl of proteinase K (200 μg/ml), and incubate in a 55 °C constant temperature incubator for 1 to 2 h. Then, incubate the product at 95 °C for 45 s, finally centrifuge at a speed of 2000 to 3000 rpm for 30 s. Store the obtained DNA sample at −20 °C for further analysis.

### Construction of Standard Recombinant Plasmids

Recombinant plasmids were prepared based on conserved sequences in the *Der f 1* (GenBank AB034946.1) and *Der p 1* (GenBank U11695.1) genes (Supplementary Table S1). The PCR reaction mixture comprises: 12.5 μl Taq polymerase, 2 μl DNA extraction solution, 1 μl each of forward and reverse primers, and 8.5 μl double-distilled water. PCR conditions are as follows: pre-denaturation at 94 °C for 5 min; 35 cycles of denaturation at 94 °C for 30 s, annealing and extension at 45 °C for 2 min; followed by a final extension at 72 °C for 10 min. Take 5 μl of PCR product and mix with 1 μl of 6× DNA loading buffer. Detect the brighter target band via 1% agarose gel electrophoresis, then purify and recover the DNA using an agarose gel DNA recovery kit. And enzymatic cleavage was initially performed, with subsequent connection into a pUC vector (Beijing Tsingke Biotechnology, Beijing, China). It was then transformed into *Escherichia coli* TOP10 competent cells, and the positive plasmids were screened by blue-white screening. The resultant positive plasmids underwent targeted sequencing using an ABI 3730 sequencer. Recombinant plasmids containing sequences from *D. farinae* and *D. pteronyssinus* were individually quantified using multiplexed microtiter digital PCR (dPCR) (Pilot Genetics Technology Co. Ltd., Hangzhou, China).

### Quantification of Plasmid Concentration

Based on the conserved sequences of the published *Der f 1* and *Der p 1* gene sequences, specific primers and probes for the *Der f 1* and *Der p 1* genes were designed, respectively, and plasmid standards were quantified using a dPCR (New England Biolabs, Ipswich, Massachusetts, United States) universal kit (Supplementary Table S2). The total reaction volume for multiplex dPCR was 15 μl, consisting of 3 μl of 5 × Mix (including DNA polymerase, dNTPMix, and Buffer), 1.5 μl of both forward and reverse primers (10 μM/l), 0.5 μl of probe (1 μM), 3 μl of plasmid (10^5^ copies/μl), and sterilized ddH_2_O to reach 15 μl. The prepared reaction solutions (15 μl) were dispensed into the injection wells of a microfluidic chip (Pilot Gene Technology Co., Ltd., Hangzhou, China), followed by the addition of 10 μl of the oil phase in each well. The inlet and outlet 4 silicone caps were affixed to the microdroplet preparation instrument for microdroplet generation. Following the reaction, the microfluidic chip was cooled to room temperature and analyzed using a biochip reader to measure the concentration of the *Der f 1* and *Der p 1* plasmid standard (original plasmid dilution). The prepared chip then underwent PCR amplification with the following thermocycling conditions: pre-denaturation at 95 °C for 10 min, denaturation at 95 °C for 15 s, annealing at 58 °C for 30 s, extension at 72 °C for 60 s for 40 cycles, with a final extension at 28 °C for 60 s.

### Design of Primers, crRNA, and Probes

To enhance the efficiency of RPA isothermal amplification and utilize optimal primers for the target gene, 3 pairs of specific oligonucleotide primers were designed for *D. farinae* and *D. pteronyssinus* using online primer design software (https://www.ncbi.nlm.nih.gov/tools/primer-blast) (Table 1). Corresponding crRNA probes were developed (https://www.ncbi.nlm.nih.gov/tools/primer-blast) to target these specific sequence fragments (Supplementary Fig. S1, Table 2). DNA probes were transcribed into RNA in accordance with the guidelines provided in the manual of the HiScribe T7 Quick High Yield RNA Synthesis Kit (New England Biolabs, Ipswich, Massachusetts, United States). A T7 promoter sequence was incorporated into the 5′ end of each forward primer. The transcription products (crRNA probes) were purified using RNAXP magnetic beads, and product concentrations were quantified using a Qubit fluorometer (Thermo Fisher Scientific, Waltham, Massachusetts, United States). These recombinant plasmids were prepared as standard controls with a dilution range of 1 copies/μl to 10^9^ copies/μl and stored at −20 °C for further utilization. All primers used in this study were synthesized by Beijing Tsingke Biotechnology (China), and RNAXP magnetic beads were provided by Vazyme Biotech Co., China.

**Table 1.** Primers used for primary screening of *Dermatophagoides farinae* and *Dermatophagoides pteronyssinus*

	Name	Sequence (5′–3′)	Amplicon size (bp)	Slope	Efficiency (%)
**Primer 1**	D. f-F1	GAAATTAATACGACTCACTATAGGG TTGGAATCATTGAAATATGTTGAAGCTAAC	112	∼−3.35	∼98
	D. f-R1	GTTCAAAAGCTTCAGCACTCATCAAATAAC			
**Primer 2**	D. f-F2	GAAATTAATACGACTCACTATAGGG TATTGGCATTAAAGATTTGAGAGCTTTTC	159	∼−3.38	∼97
	D. f-R2	TATCCCAACTGTTTCGTACGATCCAATAAT			
**Primer 3**	D. f-F3	GAAATTAATACGACTCACTATAGGG GGTATCTCAAACTACTGCCAAATTTATCCA	142	∼−3.32	∼100
	D. f-R3	TTGATTGTTCGTCCATCATAATGTTGAAAAG			
**Primer 4**	D. p-F4	GAAATTAATACGACTCACTATAGGG CCAAATTTACCCACCAAATGTAAACAAAAT	100	∼−3.40	∼96
	D. p-R4	GAATGCGTCTAAATCTTTGATGCCAATAAT			
**Primer 5**	D. p-F5	GAAATTAATACGACTCACTATAGGG AATTTACCCACCAAATGTAAACAAAATTCG	106	∼−3.36	∼98
	D. p-R5	ATAATGACGGAATGCGTCTAAATCTTTGAT			
**Primer 6**	D. p-F6	GAAATTAATACGACTCACTATAGGG GTATCTCAAACTACTGCCAAATTTATCCAC	123	∼−3.45	∼94
	D. p-R6	AATGTTGAAAAGCTCTCAAATCTTTAATGC			
**T7promoter**		GAAATTAATACGACTCACTATAGGG			

**Table 2.** Primers, probes, and RNA reporter molecules used in this study

	Name	Sequence (5′–3′)
**Primer 3**	D. f-F3	GGTATCTCAAACTACTGCCAAATTTATCCA
	D. f-R3	TTGATTGTTCGTCCATCATAATGTTGAAAAG
**Primer 4**	D. p-F4	CCAAATTTACCCACCAAATGTAAACAAAAT
	D. p-R4	GAATGCGTCTAAATCTTTGATGCCAATAAT
**T7 promoter**		GAAATTAATACGACTCACTATAGGG
**Probe-D. f**	Probe-D. f-a	TAGCGCTGTGGGTTTGAGCCAAAGCTTCAC
	Probe-D. f-b	AAATCCCCTATAGTGAGTCGTATTAATTTC
**Probe-D. p**	Probe-D. p-a	GAGTCAAAGCTTCACGGATTTGTTTCAC
	Probe-D. p-b	ATCCCCTATAGTGAGTCGTATTAATTTC
**RNA reporter molecule**		FAM-mArArUrGrGr CmAmArArUrGrGrCmA-Bio

All the 5′ ends of the forward primers in the table have integrated the T7 promoter sequence (GAAATTAATACGACTCACTATAGGG), which provides template support for RNA transcription. This promoter sequence is not a standalone primer but part of the forward primer.

### Establishment of RPA–Cas13a–LFD Detection System

Real-time detection of the target was achieved through a targeted gene-editing technique that leveraged Cas13a-mediated collateral cleavage of reporter RNA in conjunction with nucleic acid amplification. Fragments of *Der f 1* and *Der p 1* were selectively amplified using RPA. The total volume of qPCR reaction was 25 μl, containing 12 μl of Hieff qPCR SYBR Green Master Mix (Lesun Biotechnology Co., Ltd., Wuxi, China), 1 μl of plasmid (10^5^ copies/μl), 2 μl each of upstream and downstream primers (10 μM/l), and 8 μl of pure water, Highly significant correlation (*R*^2^ > 0.99), with slope values within an acceptable range (between −3.1 and −3.6) (Table 1). The release of fluorescent signals was mediated by Cas13a collateral RNA cleavage upon detecting RNA that had been transcribed and amplified from DNA targets by T7 RNA polymerase. The LFD usually contains 2 lines: The first line is the control line (C line), which contains streptavidin and can bind to biotin groups in the reporting group; The second line is the testing line (T-line), which contains 6-FAM antibodies that can bind to FAM (6-carboxyfluorescein) groups in the RNA reporter molecule. FAM is a commonly used fluorescent dye in molecular biology. Its excitation wavelength is 495 nm, and its emission wavelength is 520 nm. RNA probes labeled with FAM achieve signal visualization through side-stream detection during the detection process, aiding in accurately determining the presence of target fragments.

### RPA–Cas13a–LFD Detection System

The primary RPA reaction was executed using a thermostatic nucleic acid amplification kit (Lesun Biotechnology Co., Ltd., Wuxi, China). The RPA reaction volume totaled 47.5 μl, comprising 25 μl of reaction buffer, 17.5 μl of pure water, 2 μl of the upstream primer (10 nM/l) (Table 2), 2 μl of the downstream primer (10 nM/l), and 1 μl of plasmid (10^5^ copies/μl), the concentrations of the *Der f 1* gene plasmid standard (10^6^-fold dilution of stock solution) and *Der p 1* gene plasmid standard (10^6^-fold dilution of stock solution) were 2.23 × 10^9^ and 3.97 × 10^9^ copies/μl, respectively. The primers used for quantitative analysis of plasmid concentration can be found in Supplementary Table S2. The reaction mixture was combined with RPA lyophilized powder and mixed well, followed by adding 2.5 μl of the initial reaction mixture into the tube cap to initiate the reaction conditions were set to 39 °C in the water bath for 35 min, with brief centrifugation at the 5-min mark before returning to the water bath.

Subsequently, T7 transcription and Cas13a shearing reactions were performed in the RPA lyophilized powder tubes. Specifically, 1 μl of RPA reaction product was added to the tubes, followed by the addition of 2 μl of 10×  Cas buffer (Guangzhou Magigen Biotechnology Co., Ltd., Guangzhou, China), 1 μl of LwCas13a protein (1pM) (Guangzhou Magigen Biotechnology Co., Ltd., Guangzhou, China), 0.5 μl of RNA reporter molecule (1 μM) (Integrated DNA Technologies, Coralville, Iowa, United States), 0.5 μl of RNase inhibitor (40 U/μl) (New England Biolabs), 1 μl of crRNA (50 ng/μl), and 0.6 μl of 10× reaction buffer (Vazyme Biotech Co., China.), 1 μl T7 RNA polymerase (50 U/μl) (Vazyme Biotech Co., China.), and 1 μl dNTP (100 mM/μl) (Vazyme Biotech Co., China.). The volume of the mixture was adjusted to 20 μl using RNase-free water and incubated at 37 °C for 20 min.

Following the T7 transcription and Cas13a cleavage steps, 5.0 μl of the Cas reaction solution was diluted with 45 μl of RNase-free water and applied to lateral flow test strips (Gu’an Beiji Biotechnology Co., Ltd., Hebei, China). Observations were made after a 2-min interval.

### Optimization of RPA–Cas13a–LFD Reaction System, Detection of Specificity and Sensitivity

For the RPA system, this study involved purifying and electrophoresing the amplified products, comparing fragment sizes to ascertain whether they were the desired amplified products, and determining the suitable temperature and time based on amplification results and fragment sizes. For the Cas system, we selected the optimal concentration of RNA reporter molecules, LwCas13a protein concentration, crRNA input, and duration based on the test results of the test strips.

To verify the specificity of the RPA–Cas13a–LFD reaction system targeting the *Der f 1* and *Der p 1* genes, genomic DNA was extracted from *Aleuroglyphus ovatus*, *Tyrophagus putrescentiae*, *Blomia tropicalis*, *Suidasia nesbitti*, and *Carpoglyphus lactis* and used as control templates in parallel with the RPA–Cas13a–LFD assay. Positive plasmids containing the *Der f 1* and *Der p 1* genes served as positive controls, while RNase-free water acted as the negative control. This further assessed the specificity of the RPA–Cas13a–LFD method for detecting the *Der f 1* gene of *D. farinae* and the *Der p 1* gene of *D. pteronyssinus*. To validate the sensitivity of the RPA–Cas13a–LFD detection, the *Der f 1* and *Der p 1* positive plasmids underwent serial 10-fold dilutions. The plasmid gradient dilution copy numbers ranged from 10^6^ to 10^−1^ copies/μl, encompassing 10^6^, 10^5^, 10^4^, 10^3^, 10^2^, 10, 1, and 0.1 copies/μl.

### Evaluation of RPA–Cas13a–LFD Detection Method

To validate the efficacy of the RPA–Cas13a–LFD assay, we used the RAND function to take 20 repeatable positive integers at random between 1 and 20 and kept a record (Supplementary Table S3). To detect *D. farinae* and *D. pteronyssinus*, 40 dust samples were collected, 20 of each weighing 100 mg. These dust samples were sequentially assigned and assayed according to the numbers listed in Supplementary Table S3 to ensure consistent handling and processing methods for both species. Genomic DNA was extracted from dust samples using a Rapid Soil DNA Isolation Kit (B518233-0050, Sangon, China). Subsequently, DNA was extracted from the samples and analyzed using the established RPA–Cas13a–LFD assay. These results were then compared with those obtained through qPCR and ELISA analyses. We used Der f 1 and Der p 1 ELISA 2.0 kits (INDOOR, Biotechnologies, Ltd, Manchester, United Kingdom) to detect allergen levels. For Der f 1 and Der p 1, the sample’s LOD was set at 0.19 and 0.78 ng/ml, respectively; based on this LOD, samples below this value were negative.

## Results

### Primer Screening Results

**Fig. 1.**
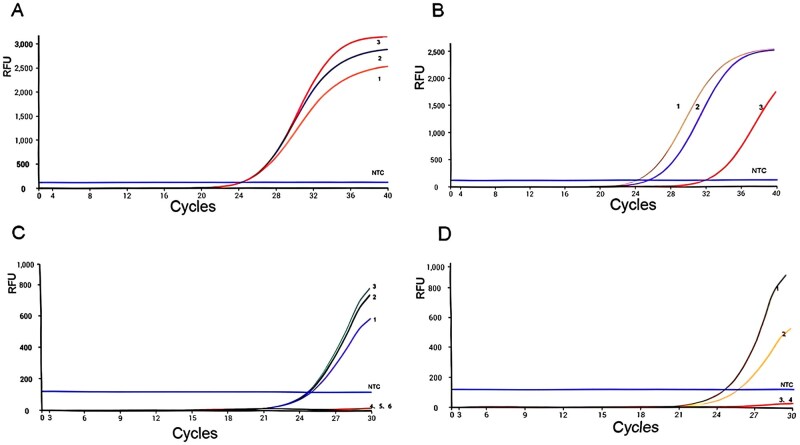
Real-time fluorescence PCR curve. (A) 1: *Dermatophagoides farinae* plasmid + *D. farinae* primer 1; 2: *D. farinae* plasmid + *D. farinae* primer 2; 3: *D. farinae* plasmid + *D. farinae* primer 3; NTC: ddH_2_O. (B) 1: *D. pteronyssinus* plasmid + *D. pteronyssinus* primer 4; 2: *D. pteronyssinus* plasmid + *D. pteronyssinus* primer 5; 3: *D. pteronyssinus* plasmid + *D. pteronyssinus* primer 6; NTC: ddH_2_O. (C) 1: *D. farinae* plasmid + *D. farinae* primer 1; 2: *D. farinae* plasmid + *D. farinae* primer 2; 3: *D. farinae* plasmid + *D. farinae* primer 3; 4: *D. pteronyssinus* plasmid + *D. farinae* primer 1; 5: *D. pteronyssinus* plasmid + *D. farinae* primer 2; 6: *D. pteronyssinus* plasmid + *D. farinae* primer 3; NTC: ddH_2_O. (D) 1: *D. pteronyssinus* plasmid + *D. pteronyssinus* primer 4; 2: *D. pteronyssinus* plasmid + *D. pteronyssinus* primer 5; 3: *D. farinae* plasmid + *D. pteronyssinus* primer 4; 4: *D. farinae* plasmid + *D. pteronyssinus* primer 5; NTC: ddH_2_O.

Primer efficacy was evaluated through qPCR, as shown in Fig. 1. Primers 1, 2, and 3 were effective in amplifying the target gene (Fig. 1A); however, only primers 4 and 5 were successful for *D. pteronyssinus* (Fig. 1B). According to Fig. 1B, primer pair 6 was wholly excluded from further testing, especially considering the high sequence homology (85.54%) in allergen genes between *D. farinae* and *D. pteronyssinus*. Based on the data presented, primer 3 was selected for *D. farinae* and primer 4 for *D. pteronyssinus* (Fig. 1C and D). These primers were subsequently employed for probe design, as detailed in Table 2.

### RPA–Cas13a–LFD Detection System

**Fig. 2.**
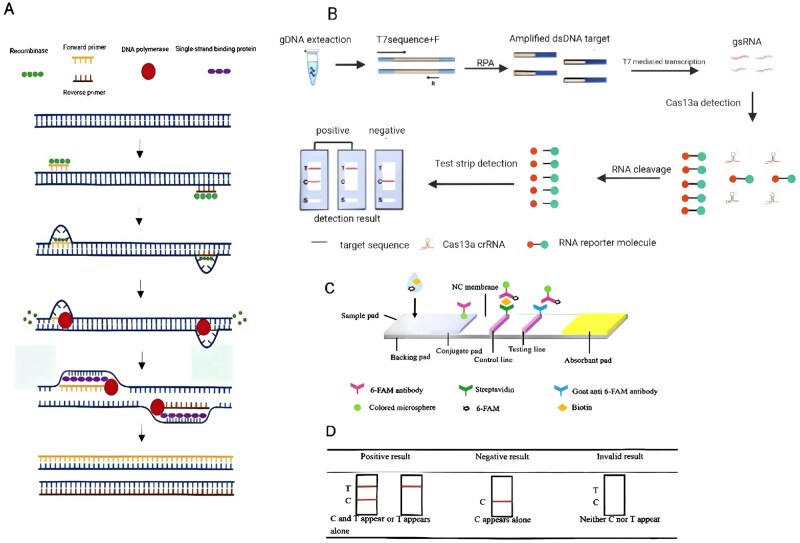
Principle diagram. (A) Schematic of the mechanism of RPA. (B) Schematic of RPA and LFD workflow coupled with CRISPR-Cas13a (RPA–Cas13a–LFD) system. (C) Structural diagram of LFD. (D) Interpretation of RPA–Cas13a–LFD assay outcomes. T, testing line; C, control line.

When a target fragment is absent in the detection process, the RNA reporter molecule is not activated, the Cas protein is not activated, and the biotin groups can bind to streptavidin in the C line. At the same time, FAM binds to the 6-FAM antibody, causing the colored microsphere to remain in the C line and display bands; in the T line, there are no bands. However, when a target fragment is present, Cas protein is activated, the RNA reporter molecule is activated, and Biotin can still bind to streptavidin in the C line while binding to goat anti mouse antibody. The colored microspheres remain in the T line and display the bands as a result of the separation of FAM and Biotin; if the RNA reporter molecule is completely cleaved, then there is no band in the C line; if the RNA reporter molecule is partially cleaved, then there are bands in the C line and the T line at the same time. A scheme of the reaction and analysis procedure is shown in Fig. 2A–C, with the final detection outcomes illustrated (Fig. 2D).

### Optimization, Specificity, and Sensitivity Evaluation of Detection System

**Fig. 3.**
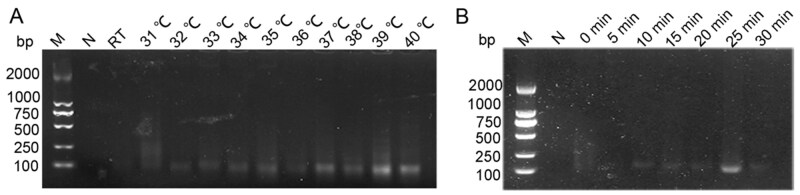
Optimization result of RPA system under different conditions for *Dermatophagoides farinae*. (A) Electrophoresis images of RPA system under different temperatures. Arrangement order: negative control (ddH_2_O), 25 °C (room temperature), 31, 32, 33, 34, 35, 36, 37, 38, 39, and 40 °C. (B) Electrophoresis images of RPA system at different times. Arrangement order: negative control (ddH_2_O), 0, 5, 10, 15, 20, 25, and 30 min. M, marker; N, negative control; RT, room temperature.

The results confirmed that the optimal temperature of 39 °C, as recommended by the kit, ensured the highest detection efficiency and was the most suitable for achieving reliable amplification in this study (Fig. 3A). In the time optimization experiments, the results indicated superior amplification efficacy at 25 min. By these findings, 25 min was selected as the duration for the RPA reactions (Fig. 3B).

**Fig. 4.**
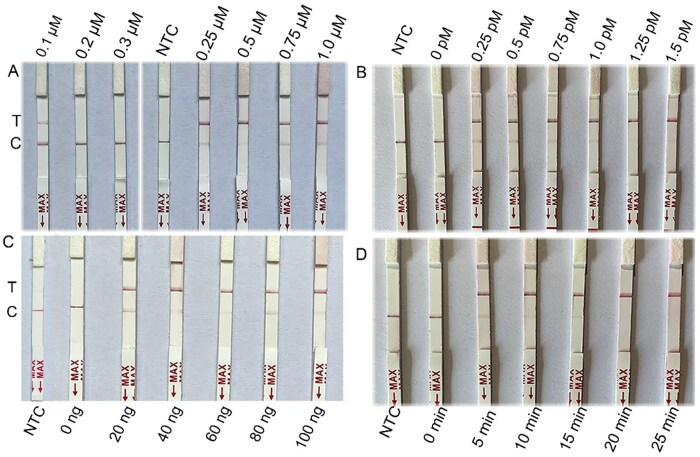
Optimization results of Cas system under different conditions for *Dermatophagoides farinae*. (A) Test strip results of Cas system at different RNA reporter concentrations. Arrangement order: 0.1, 0.2, 0.3 μM, negative control (ddH_2_O), 0.25, 0.5, 0.75, 1.0 μM. (B) Test strip results of Cas system at different LwCas13a protein concentrations. Arrangement order: negative control (ddH_2_O), 0, 0.25, 0.5, 0.75, 1.0, 1.25, and 1.5 pM. (C) Test strip results of Cas system under different crRNA inputs. Arrangement order: negative control (ddH_2_O), 0, 20, 40, 60, 80, and 100 ng. (D) Test strip results of Cas system at different times. Arrangement order: negative control (ddH_2_O), 0, 5, 10, 15, 20, and 25 min. NTC, negative control (ddH_2_O); T, test line; C, control line.

For RNA reporter concentration optimization, results revealed that a concentration of 0.1 μM led to the appearance of false positives (Fig. 4A). However, the testing line was evident starting from the second test strip (Fig. 4A and Supplementary Fig. S2A). Thus, to mitigate the risk of false positives associated with low concentrations, a concentration of 0.5 μM was selected as optimal. For LwCas13a protein concentration optimization, a concentration of 1 pM resulted in the appearance of the testing line but not the control line (Fig. 4B and Supplementary Fig. S2B), suggesting complete cleavage of the RNA reporter molecule. Thus, 1 pM was selected as the optimal concentration for the LwCas13a protein. For crRNA input optimization, a concentration of 20 ng led to the appearance of the testing line (Fig. 4C and Supplementary Fig. S2C). As such, 20 ng was selected as the optimal input amount for the crRNA. For reaction incubation time optimization, a duration of 5 min led to the appearance of the testing line (Fig. 4D and Supplementary Fig. S2D). To ensure the reliability of detection, thus, 10 min was selected as the reaction incubation time for the Cas system.

**Fig. 5.**
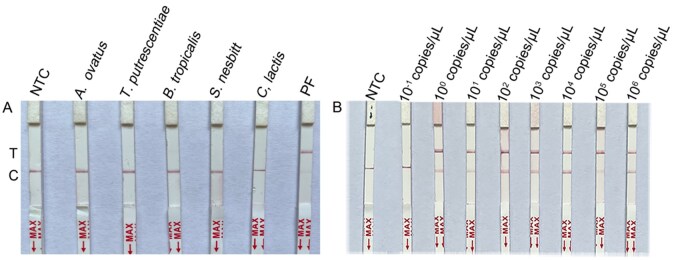
Specificity and sensitivity detection of RPA and LFD coupled with CRISPR-Cas13a (RPA–Cas13a–LFD) of *Dermatophagoides farinae*. (A) Specificity detection. 1: *Aleuroglyphus ovatus*; 2: *Tyrophagus putrescentiae*; 3: *Blomia tropicalis*; 4: *Suidasia nesbitt; 5: Carpoglyphus lactis*. (B) Sensitivity detection. *D. farinae* plasmid copy number diluted to ddH_2_O, 10^−1^, 10^0^, 10^1^, 10^2^, 10^3^, 10^4^, 10^5^, and 10^6^ copies/μl. NTC, negative control (ddH_2_O); PF, positive plasmid of *D. farinae*; T, test line; C, control line.

The assay demonstrated no cross-reactivity between *D. farinae* and *D. pteronyssinus* and other mite species, thus indicating high specificity (Fig. 5A and Supplementary Fig. S3A).

The initial concentration for the *D. farinae* plasmid standard was established as 2.23 × 10^9^ copies/μl. Results indicated that a chromogenic testing line was detected within the concentration range of 2.23 × 10^0^ to 2.23 × 10^6^ copies/μl, indicating that the LOD for *D. farinae* in the RPA–Cas13a–LFD assay was 2.23 copies/μl (Fig. 5B).

Similarly, the initial concentration for the *D. pteronyssinus* plasmid standard was 3.97 × 10^9^ copies/μl. Results revealed that a colored testing line was detected within the concentration range of 3.97 × 10^1^ to 3.97 × 10^6^ copies/μl, indicating that the LOD for *D. pteronyssinus* in the RPA–Cas13a–LFD assay was 39.7 copies/μl (Supplementary Fig. S3B).

### Assessment of RPA–Cas13a–LFD Assay

**Fig. 6.**
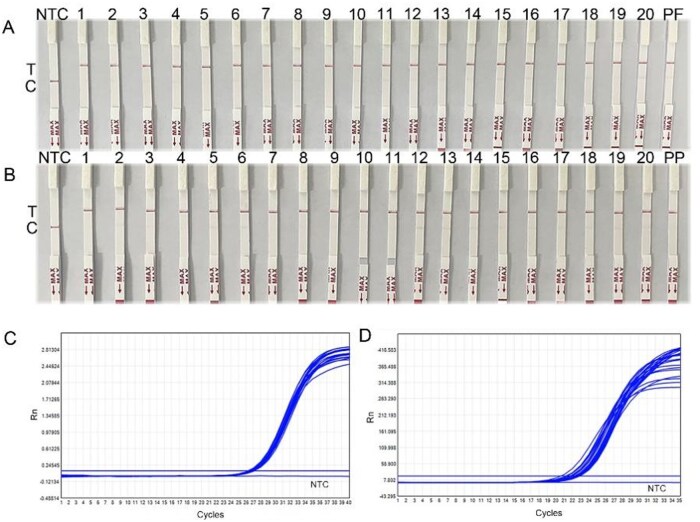
Assessment of RPA and LFD coupled with CRISPR-Cas13a (RPA–Cas13a–LFD). (A) The results for *Dermatophagoides farinae*. (B) The results for *Dermatophagoides pteronyssinus*; Curves for qPCR. (C) The results for *D. farinae*. (D) The results for *D. pteronyssinus*. NTC, negative control (ddH_2_O); PF, positive plasmid of *D. farina*; T, test line; C, control line.

Within the set of examined samples for *D. farinae*, RPA–Cas13a–LFD and qPCR identified 20 positive samples (Fig. 6A and C), while ELISA identified 19 positive samples and 1 negative sample (Supplementary Table S4). Thus, the concordance rate between the 2 methods was 95%, and the disparities in their respective outcomes were insignificant (*p *> 0.05, Supplementary Table S5).

Within the set of examined samples for *D. pteronyssinus*, RPA–Cas13a–LFD and qPCR identified 20 positive samples (Fig. 6B and D), while ELISA identified 18 positive samples and 2 negative samples (Supplementary Table S6). Thus, the concordance rate between the 2 methods was 90%, and the disparities in their respective outcomes were insignificant (*p *> 0.05, Supplementary Table S7).

In the case of the *Der f 1* gene of *D. farinae* and the *Der p 1* gene of *D. pteronyssinus*, the concordance rates were 100% (20/20) both by RPA–Cas13a–LFD and qPCR, which were higher than the concordance rates of 95% (19/20) and 90% (18/20) by the ELISA.

## Discussion

An ideal diagnostic approach for infection identification should be instrument-free, sensitive, specific, rapid, and cost-effective (Wang et al. 2020). In this study, we developed a novel RPA and CRISPR-Cas13a system for detecting dust mite for the first time, with results easily observable using LFD. Our combined method demonstrated a higher detection rate compared to the single-tube approach previously described by Wang et al. (2019), with both RPA and Cas13a reactions conducted simultaneously. Furthermore, our method achieved a detection time of 35 min and exhibited detection limits of 2.23 and 10^2^ copies/μl (detection limit of 2.23 copies/μl for *D. farinae* and 39.7 copies/μl for *D. pteronyssinus*), with no cross-reactivity with other mite species.

Conventional nucleic acid-based techniques for detecting dust mites, such as traditional PCR, nested PCR, and qPCR, offer high sensitivity but are encumbered by complex equipment requirements and operator expertise, limiting their suitability for on-site application. In contrast, our RPA–Cas13a–LFD detection system enhanced the sensitivity and specificity of pathogen detection, with LFD-based visualization found to be suitable rapidly detecting dust mite. Currently, ELISA is the most widely used technique for quantifying dust mite allergens (Wood et al. 1988). Commercial dual-site monoclonal antibody ELISA kits use non-isotopic immunoassays to detect major inhaled allergens *Der f 1* and *Der p 1*, achieving a detection sensitivity of 10^−1 ^ng/μl by targeting shared epitopes (Luczynska et al. 1989). However, cross-allergens’ prevalence among mite species renders protein-based detection methods susceptible to false positives arising from cross-reactions. Xue et al. (2023) established a LAMP-based approach for detecting *D. farinae*, with an LOD of 10^−2 ^ng/μl, while Thet-Em et al. (2012) established a multiplex PCR system for detecting *D. farinae*, *D. pteronyssinus*, and *Blomia tropicalis* with an LOD of 10^0^ ng/μl.

In contrast, our newly developed RPA–Cas13a–LFD system requires only 10^0^–10^2^ copies/μl to detect *D. farinae* and *D. pteronyssinus*, demonstrating superior sensitivity and potential for rapid diagnostics. This study used RPA–Cas13a–LFD, qPCR, and ELISA to detect dust mites in selected areas of Wuhu in China. The detection rates based on the RPA–Cas13a–LFD and qPCR methods were consistent (100%) and surpassed that achieved by ELISA (95% and 90% for *D. farinae* and *D. pteronyssinus*, respectively). However, potential biases may exist due to the limited sample size in this validation study. Furthermore, our study was restricted to the analysis of *Der f 1* and *Der p 1* genes, omitting other allergens and possibly introducing inaccuracies. Therefore, additional studies are ­necessary for further validation.

In conclusion, we successfully established a novel RPA–Cas13a–LFD-based method for rapidly detecting dust mite. This approach satisfies essential criteria, including high specificity and sensitivity, rapid assay completion, and straightforward visualization without the need for complex and expensive equipment. This technique holds promise for the screening of human residential environments as well as clinical evaluations of dust mite. In addition to the DNA extraction steps required in the laboratory, RPA amplification, T7 transcription, and Cas13a cleavage reactions have all been pre-integrated into a single-tube operation, with signal visualization achieved through labeling RNA probes with FAM. The detection process only requires adding the sample to the reaction tube and placing it in a water bath to initiate the reaction. Subsequently, the characteristic bands showing the dT line and C line can determine the detection result. This design simplifies the complex steps of multiple reagent additions and makes this method suitable for convenient on-site operation by nonprofessionals. The future research direction of this study is to simplify the operation process further. We will continue to optimize and integrate this detection method to make all reaction steps more automated, enabling nonprofessional users to quickly complete the detection on-site, while retaining the advantages of higher detection rate and shorter detection time that we currently possess. This integrated solution is the ultimate goal of this research.

## Supplementary Material

ieag029_Supplementary_Data
