# Supplementary material for: A novel rapid detection approach based on CRISPR-Cas13a for Dermatophagoides pteronyssinus and Dermatophagoides farinae (Acariformes: Pyroglyphidae)
Source: J Insect Sci. 2026 Apr 18;26(2):ieag029. doi: 10.1093/jisesa/ieag029 (PMC13091095; doi:10.1093/jisesa/ieag029)
Supplement: ieag029_Supplementary_Data [file ieag029_supplementary_data.zip › 06-Apr-2026_082941_Supplement_Data.zip!/supplementary__materials_figures and tables.docx1.docx]

**Figure S1.** Target sequence-aligned crRNA CRISPR-Cas13a gene editing mechanism.

**Figure S2.** Optimization results of Cas system under different conditions for *Dermatophagoides pteronyssinus*.(A) Test strip results of Cas system at different RNA reporter concentrations. Arrangement order: negative control (ddH2O),0.25 μM, 0.5 μM, 0.75 μM, 1.0 μM.(B) Test strip results of Cas system at different LwCas13a protein concentrations. Arrangement order: negative control (ddH2O), 0 pM, 0.25 pM, 0.5 pM, 0.75 pM, 1.0 pM, 1.25 pM, and 1.5 pM.(C) Test strip results of Cas system under different crRNA inputs. Arrangement order: negative control (ddH2O), 0 ng, 20 ng, 40 ng, 60 ng, 80 ng and 100 ng.(D) Test strip results of Cas system at different times. Arrangement order: negative control (ddH2O),0 min, 5 min, 10 min, 15 min, 20 min and 25 min.

**Figure S3.** Specificity and sensitivity detection of RPA and LFD coupled with CRISPR-Cas13a (RPA-Cas13a-LFD) of *Dermatophagoides pteronyssinus*. (A) Specificity detection. 1: *Aleuroglyphus ovatus*; 2: *Tyrophagus putrescentiae*; 3: *Blomia tropicalis*; 4: *Suidasia nesbitt; 5: Carpoglyphus lactis*. (B) Sensitivity detection. *D. pteronyssinus* plasmid copy number diluted to ddH_2_O, 10^-1^, 10^0^, 10^1^, 10^2^, 10^3^, 10^4^, 10^5^, and 10^6^ copies/μL. NTC: negative control (ddH_2_O); PF: Positive plasmid of *D. farinae*; T: Test line; C: Control line.

**Figure S1.** Target sequence-aligned crRNA CRISPR-Cas13a gene editing mechanism.

**Figure S2.** Optimization results of Cas system under different conditions for *Dermatophagoides pteronyssinus*.(A) Test strip results of Cas system at different RNA reporter concentrations. Arrangement order: negative control (ddH2O),0.25 μM, 0.5 μM, 0.75 μM, 1.0 μM.(B) Test strip results of Cas system at different LwCas13a protein concentrations. Arrangement order: negative control (ddH2O), 0 pM, 0.25 pM, 0.5 pM, 0.75 pM, 1.0 pM, 1.25 pM, and 1.5 pM.(C) Test strip results of Cas system under different crRNA inputs. Arrangement order: negative control (ddH2O), 0 ng, 20 ng, 40 ng, 60 ng, 80 ng and 100 ng.(D) Test strip results of Cas system at different times. Arrangement order: negative control (ddH2O),0 min, 5 min, 10 min, 15 min, 20 min and 25 min.

**Figure S3.** Specificity and sensitivity detection of RPA and LFD coupled with CRISPR-Cas13a (RPA-Cas13a-LFD) of *Dermatophagoides pteronyssinus*. (A) Specificity detection. 1: *Aleuroglyphus ovatus*; 2: *Tyrophagus putrescentiae*; 3: *Blomia tropicalis*; 4: *Suidasia nesbitt; 5: Carpoglyphus lactis*. (B) Sensitivity detection. *D. pteronyssinus* plasmid copy number diluted to ddH_2_O, 10^-1^, 10^0^, 10^1^, 10^2^, 10^3^, 10^4^, 10^5^, and 10^6^ copies/μL. NTC: negative control (ddH_2_O); PF: Positive plasmid of *D. farinae*; T: Test line; C: Control line.

**Table S1.** Primers used in PCR

| Name | Sequences (5‘-3’) |
| --- | --- |
| D.f-dF | GGATCCATGAAATTCGTTTTGGCCATTGCT |
| D.f-dR | CTCGAGTTAGATGACAATTTTAACAGCTGG |
| D.p-dF | GGAATTCCATATGAAATTGTCTCTCGCTGTTGC |
| D.p-dR | CCGCTCGAGTCAGATGACAATTTTGACAGCTAC |

**Table S2.** Primers and probes used in dPCR

| Name | Sequences (5‘-3’) |
| --- | --- |
| D.f-dF | GGTATCTCAAACTACTGCCAAATTTATCCA |
| D.f-dR | TTGATTGTTCGTCCATCATAATGTTGAAAAG |
| D.f-probe | CCAGATGTGAAACAAATCCGTGAAGCTTTGACTCAAACACACACAGCTATTGCCGTCATTATTGGCATTAAAGATTTGAGAG |
| D.p-dF | CCAAATTTACCCACCAAATGTAAACAAAAT |
| D.p-dR | GAATGCGTCTAAATCTTTGATGCCAATAAT |
| D.p-probe | TCGTGAAGCTTTGGCTCAAACCCACAGCGCTATTGCCGTC |

**Table S3.** Place 20 randomly selected samples into different numbers of mites

| Number | 1 | 2 | 3 | 4 | 5 | 6 | 7 | 8 | 9 | 10 | 11 | 12 | 13 | 14 | 15 | 16 | 17 | 18 | 19 | 20 |
| --- | --- | --- | --- | --- | --- | --- | --- | --- | --- | --- | --- | --- | --- | --- | --- | --- | --- | --- | --- | --- |
| Mite count | 16 | 20 | 10 | 2 | 6 | 19 | 6 | 9 | 9 | 11 | 16 | 13 | 7 | 18 | 18 | 5 | 3 | 10 | 16 | 8 |

**Table S4.** Concentrations of *Dermatophagoides* *farinae* samples and standards using ELISA assay

| Test | OD | Test | OD | Test | OD | Test | OD |
| --- | --- | --- | --- | --- | --- | --- | --- |
| 1 | 0.2355 | 6 | 2.0683 | 11 | 0.6858 | 16 | 1.0504 |
| 2 | 0.2214 | 7 | 0.2413 | 12 | 1.1311 | 17 | 0.1552 |
| 3 | 0.2210 | 8 | 2.0635 | 13 | 0.8412 | 18 | 0.9567 |
| 4 | 0.0524 | 9 | 2.0897 | 14 | 0.9377 | 19 | 0.9101 |
| 5 | 0.7905 | 10 | 0.6099 | 15 | 0.8723 | 20 | 0.7517 |

**Table S5.** Comparison of the results of the RPA-Cas13a-LFD method, qPCR method and ELISA method for *Dermatophagoides* *farinae*

| Methods | *D. farinae* | | | | | |
| --- | --- | --- | --- | --- | --- | --- |
|  | positive | negative | total | Testing time | Large-scale equipment | costs |
| RPA-Cas13a-LFD | 20 | 0 | 20 | 35min | NO | 3 dolar |
| qPCR | 20 | 0 | 20 | 1.5h | Yes | 4.13dolar |
| ELISA | 19 | 1 | 20 | 2h | Yes | 8.25dolar |

**Table S6.** Concentrations of *Dermatophagoides pteronyssinus* samples and standards using ELISA assay

| Test | OD | Test | OD | Test | OD | Test | OD |
| --- | --- | --- | --- | --- | --- | --- | --- |
| 1 | 0.3741 | 6 | 0.3771 | 11 | 0.4510 | 16 | 0.1452 |
| 2 | 0.1609 | 7 | 0.2743 | 12 | 0.9335 | 17 | 0.0610 |
| 3 | 0.3099 | 8 | 0.2454 | 13 | 1.2401 | 18 | 0.2533 |
| 4 | 0.3423 | 9 | 0.2718 | 14 | 0.8566 | 19 | 0.1025 |
| 5 | 0.3832 | 10 | 0.1817 | 15 | 1.0239 | 20 | 0.1464 |

**Table S7.** Comparison of the results of the RPA-Cas13a-LFD method, qPCR method and ELISA method for *Dermatophagoides pteronyssinus*

| Methods | *D. pteronyssinus* | | | | | |
| --- | --- | --- | --- | --- | --- | --- |
|  | positive | negative | total | Testing time | Large-scale equipment | costs |
| RPA-Cas13a-LFD | 20 | 0 | 20 | 35min | NO | 3 dolar |
| qPCR | 20 | 0 | 20 | 1.5h | Yes | 4.13dolar |
| ELISA | 18 | 2 | 20 | 2h | Yes | 8.25dolar |
